# Durability Analysis of CFRP Adhesive Joints: A Study Based on Entropy Damage Modeling Using FEM

**DOI:** 10.3390/ma16206821

**Published:** 2023-10-23

**Authors:** Yutong Li, Huachao Deng, Maruri Takamura, Jun Koyanagi

**Affiliations:** Department of Materials Science and Technology, Tokyo University of Science, Tokyo 125-8585, Japan; liyutong@rs.tus.ac.jp (Y.L.); dhc19911202@rs.tus.ac.jp (H.D.); 8217056@alumni.tus.ac.jp (M.T.)

**Keywords:** numerical simulation, composite laminates, entropy-based strength degradation, CFRP transverse cracking behavior, fatigue

## Abstract

Experimental methodologies for fatigue lifetime prediction are time-intensive and susceptible to environmental variables. Although the cohesive zone model is popular for predicting adhesive fatigue lifetime, entropy-based methods have also displayed potential. This study aims to (1) provide an understanding of the durability characteristics of carbon fiber-reinforced plastic (CFRP) adhesive joints by incorporating an entropy damage model within the context of the finite element method and (2) examine the effects of different adhesive layer thicknesses on single-lap shear models. As the thickness of the adhesive layer increases, damage variables initially increase and then decrease. These peak at 0.3 mm. This observation provides a crucial understanding of the stress behavior at the resin–CFRP interface and the fatigue mechanisms of the resin.

## 1. Introduction

Adhesive bonding technology has been widely developed to combine similar and dissimilar materials (glass, metals, plastics, and ceramics) owing to its many advantages over other joining methods such as fastening, riveting, brazing, and welding [1,2,3]. The technology is being used increasingly in the aerospace industry [4,5,6,7,8] owing to its superiority over conventional joining technologies in many aspects such as the specific strength, flexibility, damage tolerance, and fatigue resistance. In bonded structures, an adhesive layer is typically sprayed between two or more adherends [9]. Unlike the adherends, the adhesive layer is significantly weaker, and delamination generally occurs under cyclical mechanical or thermal loading [10,11,12]. As delamination progresses, the bond strength decreases dramatically, and the durability of the bonded joints is affected severely. Thus, an accurate fatigue lifetime estimation of the adhesive layer under cyclic loading is critical.

In previous studies, experimental methodologies were used extensively to investigate the strength and fatigue lifetime of adhesive bonding structures. Ishii et al. [13] conducted a series of fatigue tests on three types of adhesively bonded joint specimens (butt joints, scarf joints, and thick adherend lap-shear joints) to investigate the fatigue failure criteria for carbon fiber-reinforced polymer (CFRP)–metal joints under multiaxial stress conditions. Based on the two stress singularity parameters, Ishii et al. [14] proposed a method for evaluating the endurance limits of adhesively bonded single, single-cracked, and single-step double-lap joints. Ferreira et al. [15] studied the effects of layer orientation, lap joint length, and water immersion on the fatigue performance of adhesives used in PP-based composites. The results revealed that the effect of water exposure on fatigue behavior was mainly conditioned by the water temperature and, to a lesser degree, by the exposure time. Zhang et al. [16] investigated the environmental effect on the fatigue behavior of adhesively bonded pultruded joints subjected to a constant amplitude load. It was observed that the environment had a considerable effect on the fatigue behavior of the examined joints. An increased temperature appears to shorten the fatigue life of the specimen. This phenomenon is more significant in the presence of high humidity. Tang et al. [17] indicated that both static and fatigue strength values decrease with an increase in bond line thickness. They demonstrated how generalized stress intensity factors can be applied to predict fatigue failure. Recently, Reis et al. [18] investigated the effect of load frequency on the fatigue behavior of adhesively bonded steel lap joints. The results indicated that the load frequency was a key factor affecting the fatigue lifetime of the adhesive. For higher shear stress amplitudes, the frequency had a marginal effect on fatigue life. However, at lower shear stress amplitudes, fatigue life of the adhesive joints depended significantly on the frequency level. Schneider et al. [19] used the stress-life method to estimate the fatigue lifetime of joints bonded with a toughened epoxy adhesive at different temperatures. The experimental results showed that an increase in the temperature reduced the lifetime.

Considering that experimental methodologies are generally time consuming and that the results can be affected by environmental conditions such as the size of the specimen and loading conditions, researchers have been seeking efficient numerical methods for accurately predicting fatigue lifetime. Among the numerical methods used for predicting the fatigue lifetime of adhesives, the cohesive zone model (CZM) based on damage mechanics is the most widely used. Khoramishad et al. [20] developed a bilinear traction–separation description in a CZM to simulate progressive damage in adhesively bonded joints. Jimenez and Duddu [21] investigated the sensitivity of the CZM for high-cycle fatigue delamination. These sensitivity investigations showed that the separation- and strain-based fatigue damage functions were highly sensitive to cohesive stiffness and strength parameters. Fekih et al. [22] developed novel adhesive test assemblies consisting of a rigid ceramic component bonded to a resonant flexible epoxy-fiber glass (E-glass) support. Recently, the CZM has been extended to fatigue crack propagation [23] and nanocomposites [24,25]. Additionally, they determined an intrinsic fatigue damage law of adhesives. In addition to the well-known stress-based or energy-based methods for estimating fatigue life [26,27,28], approaches based on irreversible thermodynamics [29,30,31,32] have been proposed to investigate the failure mechanism and long-term lifetime of solid materials. It is widely acknowledged that both irreversible microplastic deformation and internal friction can cause permanent degradation. This is evident in plastics. From a thermodynamic perspective, these irreversible degradations can be measured by entropy. It is a nonnegative quantity that can serve as a basis for the damage evolution metric for elastic and inelastic deformations. When the entropy generation of a material attains a threshold value called fracture fatigue entropy (FFE) [33,34], final failure occurs. Several studies have reported that the estimation of fatigue life based on entropy displays potential. It should be noted that the fracture fatigue entropy of a material is also constant. This is so even in the case where A656-grade steel is subjected to ultrasonic vibration at 20 kHz [35].

Although the entropy-based failure criterion is commonly used to predict the fatigue life of metal components, its use in assessing the prolonged life of CFRP under cyclic loading is less established. Huang et al. [33] examined the influence of stacking sequences on the internal friction and fracture fatigue entropy. Additionally, the fatigue life estimation of CFRP was assessed by considering both confidence levels and reliability. Koyanagi et al. [36,37,38,39,40] recently formulated a computational approach that integrates entropy damage to analyze the failure mechanism of a viscoelastic matrix and CFRP cross-ply laminates.

This study aims to provide an understanding of the durability characteristics of CFRP adhesive joints by incorporating an entropy damage model within the context of the finite element method (FEM). FEM analyses under cyclic loading reveal that energy dissipates owing to viscosity with repeated loads, thereby causing an increase in entropy and resulting in damage. Alterations in the stress distribution are observed in relation to this process. Conventional S–N curve analyses appear to be inadequate for these observed variations. Analyses are performed for various adhesive layer thicknesses. These indicate a potential optimal thickness value.

## 2. Numerical Methodology

### 2.1. Entropy-Based Failure Criterion

**Figure 1:**
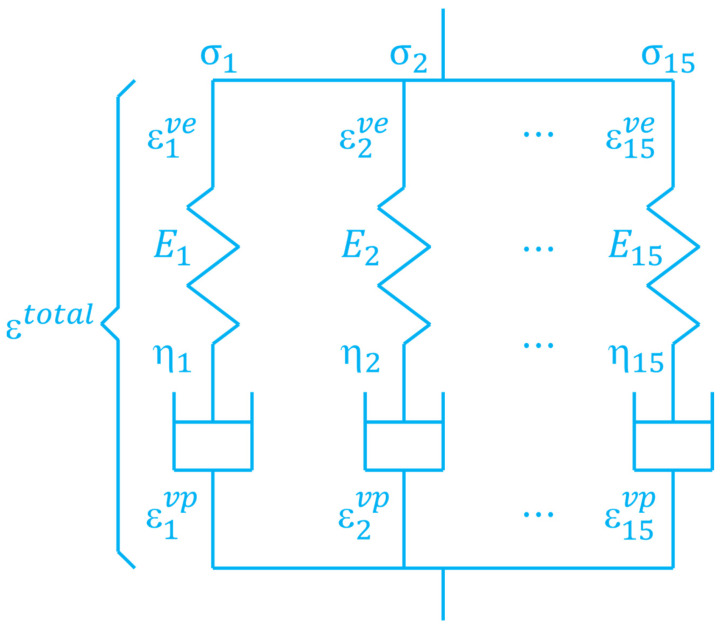
Viscoelastic model using 15 Maxwell’s elements.

In this study, the viscosity of matrix resin is addressed with the viscoelastic model using 15 Maxwell elements as shown in Figure 1. Here, the total strain ε is decomposed into the viscoelastic strain $\varepsilon^{ve}$ and viscoplastic strain $\varepsilon^{vp}$ [13]:

(1) $$\varepsilon =\varepsilon^{ve}+\varepsilon^{vp}$$

(2) $$\varepsilon^{vp}=\int_{0}^{t}H^{-1}\sigma dt$$

where σ is the Cauthy stress tensor, t is the time, and the viscosity matrix H is

(3) $$H=\frac{\eta^{vp}}{\left(1+\nu \right)\left(1-2\nu \right)}M$$

(4) $$M=\left[\begin{array}{cccccc} 1-\nu & \nu & \nu & 0 & 0 & 0\\ & 1-\nu & \nu & 0 & 0 & 0\\ & & 1-\nu & 0 & 0 & 0\\ & & & \frac{1}{2}-\nu & 0 & 0\\ & Symmetry & & & \frac{1}{2}-\nu & 0\\ & & & & & \frac{1}{2}-\nu \end{array}\right]$$

(5) $$\eta^{vp}=\eta_{0}\times \frac{1+e^{\beta {\left(\varepsilon_{eqv}^{vp}/\sigma_{eqv}\right)}^{\chi }}}{1+e^{\alpha \left(\sigma_{eqv}-\sigma_{vp0}\right)}}$$

In Equations (3)–(5), the related definitions of the material properties are adopted from [40].

A viscoelastic constitutive relationship considering the damage variable D is expressed as [41,42]:

(6) $$\sigma \left(t\right)=\left(1-D\right)\int_{0}^{t}E^{r}\left(t-t^{\prime }\right)g{\dot{\varepsilon }}^{ve}dt^{\prime }$$

where the relaxation modulus $E^{r}$ and nonlinear coefficient g are

(7) $$E^{r}\left(t\right)=\sum_{n=1}^{15}E_{ijkl}^{n}e^{-tE^{n}/\eta^{n}}$$

(8) $$g=\frac{1}{1+\alpha {\left(\frac{\sigma_{eqv}}{\sigma_{0}}\right)}^{m}}$$

In this study, entropy generation s is utilized to reveal the degradation of the matrix resin. It is calculated as

(9) $$s=\int_{0}^{t}\frac{1}{T}\sigma :{\dot{\varepsilon }}^{vp}dt$$

where T is the temperature and ${\dot{\varepsilon }}^{vp}$ is the viscoplastic rate. The final fracture entropy $s^{f}$ per unit volume of material until fracture can be calculated using Equation (9). Finally, the damage variable D related to entropy generation is defined to address the degradation of the properties as follows:

(10) $$D=\frac{s}{s^{f}}D_{cr}$$

where $D_{cr}$ is the user-defined critical damage value.

The developed entropy-based failure criterion is implemented in the user-defined subroutine of Abaqus. Elongation of the dashpot, dissipated energy increment, and other parameters are computed to obtain the increment in entropy and damage, and to update the stress components. A detailed explanation is presented in [40].

### 2.2. Finite Element Modeling

**Figure 2:**
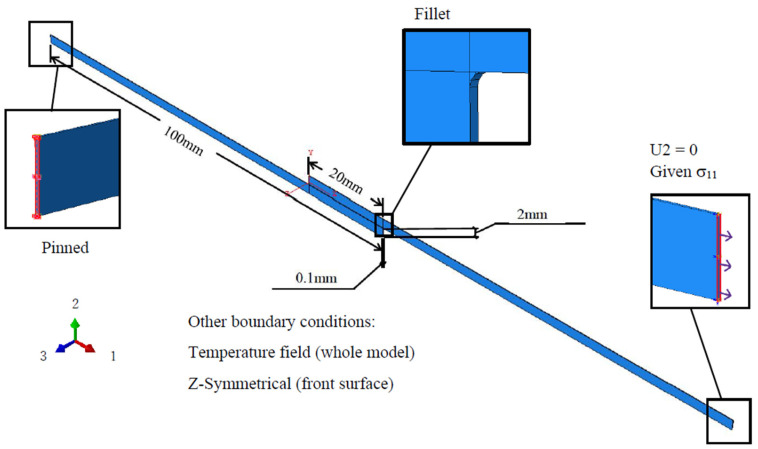
Dimension of SLS model.

In this study, Abaqus CAE 2020 (Johnston, RI, USA) is used to generate a single-lap shear (SLS) model (Figure 2). Two slabs of CFRP material each with a length of 100 mm, thickness of 2 mm, and depth of 0.1 mm are bonded in the middle using a resin with a length of 20 mm, depth of 0.1 mm, and thickness ranging from 0.1 to 0.8 mm (with a fixed increment of 0.1 mm). To mitigate the effects of mesh dependency, fillets with radius of 0.1 mm are added at the junction between the CFRP and the resin. The main body of the resin, fillet portion, and the CFRP are all assembled together using the tie type. The SLS model is pinned at the left end, whereas a cyclic sinusoidal load varying from 0 to 80 MPa at a load frequency of 2 Hz is applied from the right end. To ensure numerical stability, displacement in the Z-direction is restrained.

**Figure 3:**
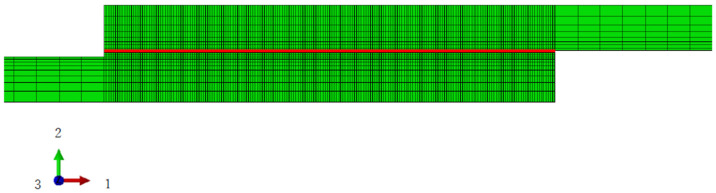
Mesh verification of resin and the collection path of stress distribution.

**Figure 4:**
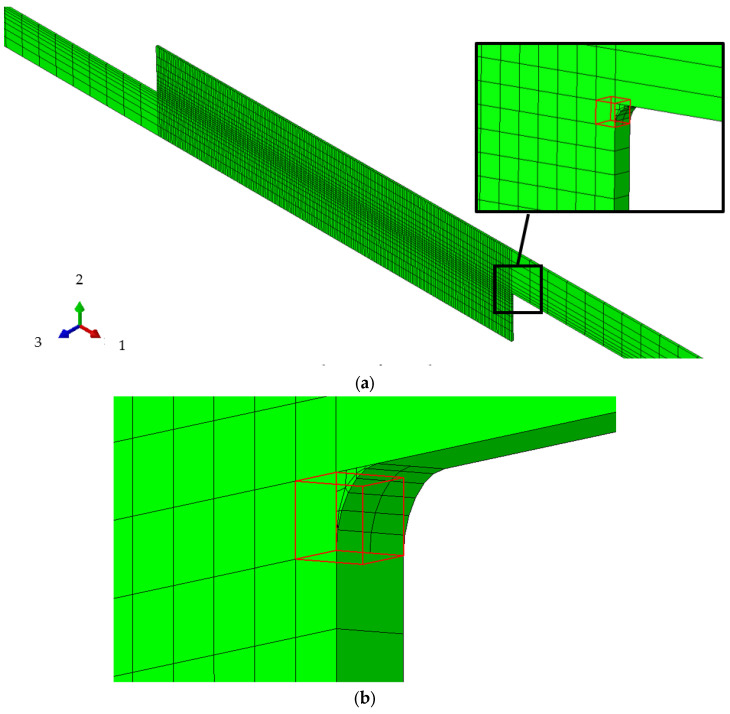
Enlarged view of elements. (**a**) Element for analysis; (**b**) Fillet mesh.

With regard to the properties of the resin, according to the study of Kagawa et al., it adopts a nonlinear, viscoelastic viscoplastic material [43]. It is implemented in Abaqus by the user subroutine UMAT. The CFRP is assumed to have orthotropic elasticity because this study focuses on the failure of resin under cyclic loading. These properties are defined by the engineering constants listed in Table 1. The detailed properties of the resin are presented in Table 2. These have been extracted from reference [40]. The original UMAT is designed for standard fatigue tests and generally requires many cycles to attain failure. To achieve fatigue failure with a limited number of tension cycles, this study introduces a parameter, **α*_d_***, into the resin’s characteristics. This serves to significantly amplify the damage incurred with each tension, thereby accelerating the completion of the simulation. The resin portion is discretized by C3D8 elements with a size of 0.1 × 0.1 × 0.1 mm. In the case of a thickness of 0.1 mm, the resin divides into a single layer of 1 × 200 elements. For the 0.2 mm model, the total number of elements in the resin is 2 × 200, and so forth. Given the rotational symmetry of stress conditions on the resin’s interfaces during tension, only the 200 elements on the upper interface of resin are considered in the following numerical analysis (Figure 3). The analytical procedure is both static and general. Additionally, because failure typically initiates from the outermost elements, only the failure behavior of the first element on the top right of the resin is discussed in this study (Figure 4).

## 3. Results and Discussions

### 3.1. Evolution of Stress along the Interface versus Cyclic Loadings

**Figure 5:**
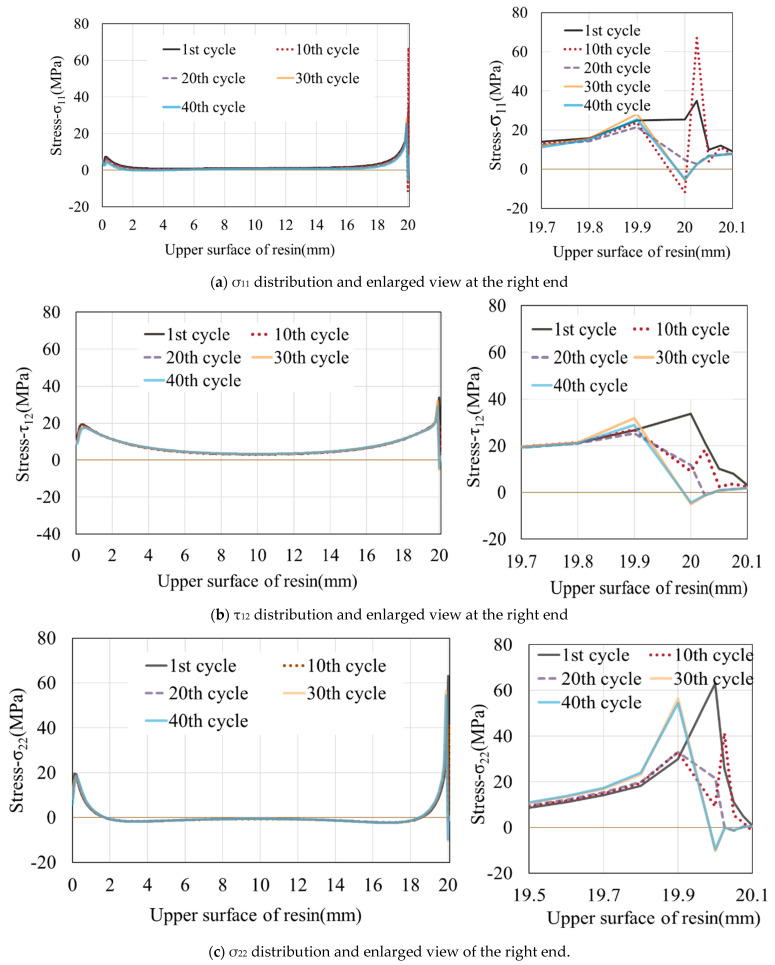
The stress distribution at different tension cycles for thickness of 0.3 mm. The data extraction locations refer to the red line in Figure 3.

Considering the model with a thickness of 0.3 mm as an example, the stress distribution at the adhesive interface (upper surface) between the resin and CFRP is shown in Figure 5. Throughout the tensile testing, the entire interface between the resin and CFRP exhibits a stress distribution. For clarity, in the subsequent analysis, we designate the leftmost point of the resin as 0 mm and rightmost extent as 20.1 mm (including the fillets). Both failure and stress peaks begin from the rightmost element and move toward the center of the resin. As the simulation progresses, the stress on a single element first increase, reaching a peak, and then gradually decreases. At the same time, the stress on the adjacent, undamaged element gradually increases. This process reflects the transfer and variation of stress among different elements. After the stress on this neighboring element attains its peak, the stress peak also shifts to this location. This process continues until all the elements fail. After the onset of tension, the stress difference in σ_11_ and σ_22_ is larger than τ_12_. However, σ_11_ is positive, whereas σ_22_ shows negative values except near its peak. This indicates that the resin experiences compression in the vertical direction.

**Figure 6:**
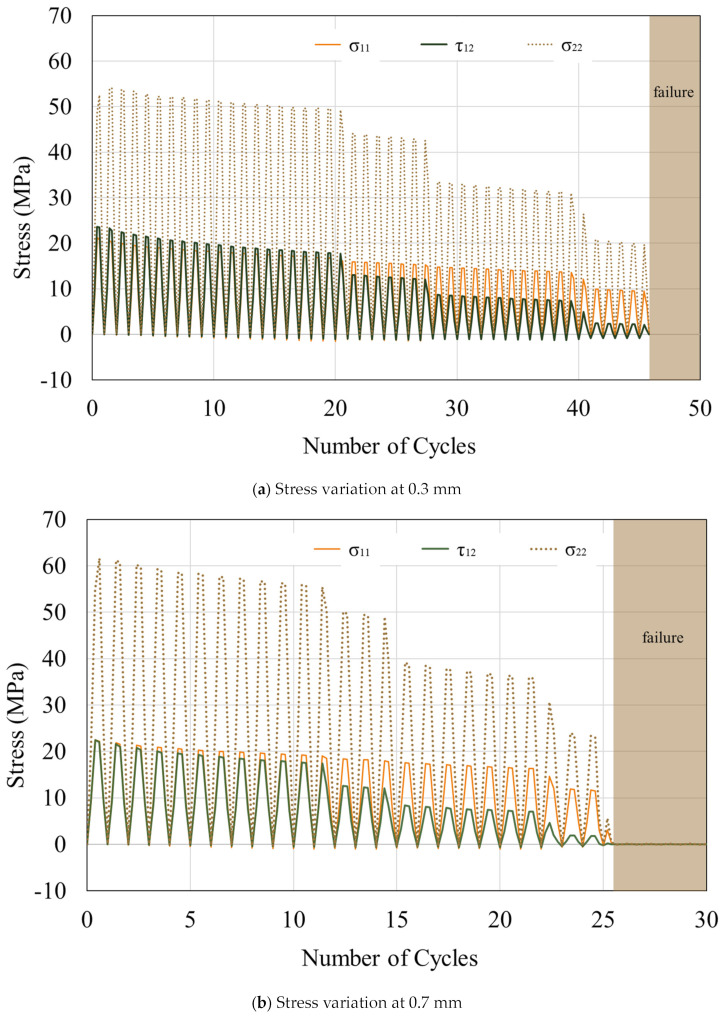
Variation in stress magnitude with number of tension cycles. The data extraction locations refer to the element highlighted by the red box in Figure 4.

When examining the centroidal stress of the uppermost right element of the resin, it can be observed that the stress values σ_11_, τ_12_, and σ_22_ reduce with an increase in the number of tension cycles (Figure 6). In an element, σ_11_ and τ_12_ are significantly smaller than σ_22_, with a ratio less than half. The values of σ_11_ and τ_12_ are initially close. However, the difference between τ_12_ and σ_11_ begins to widen when half of the failure cycles is approached. For the model with a thickness of 0.3 mm, τ_12_ > σ_11_ at the beginning of the tension, but this difference decreases as the resin thickness increases. From a thickness of 0.7 mm, σ_11_ becomes larger than τ_12_ at the beginning of the tension. This simulation indicates that even with a constant external load, the stress experienced by the resin elements during the fatigue test varies. This renders the conventional S–N curve inadequate for capturing such fatigue mechanisms. Factors other than stress should be considered when studying material fatigue failure.

### 3.2. Effect of Adhesive Thickness on the Evolution of Stress along the Interface

**Figure 7:**
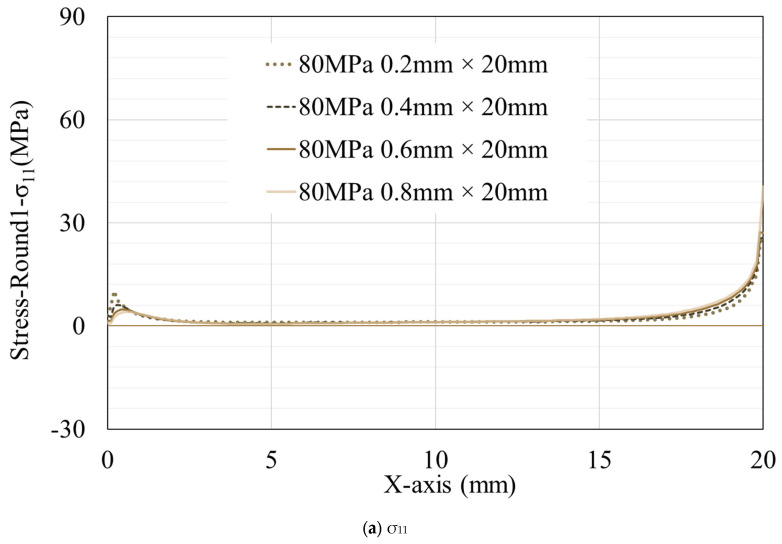

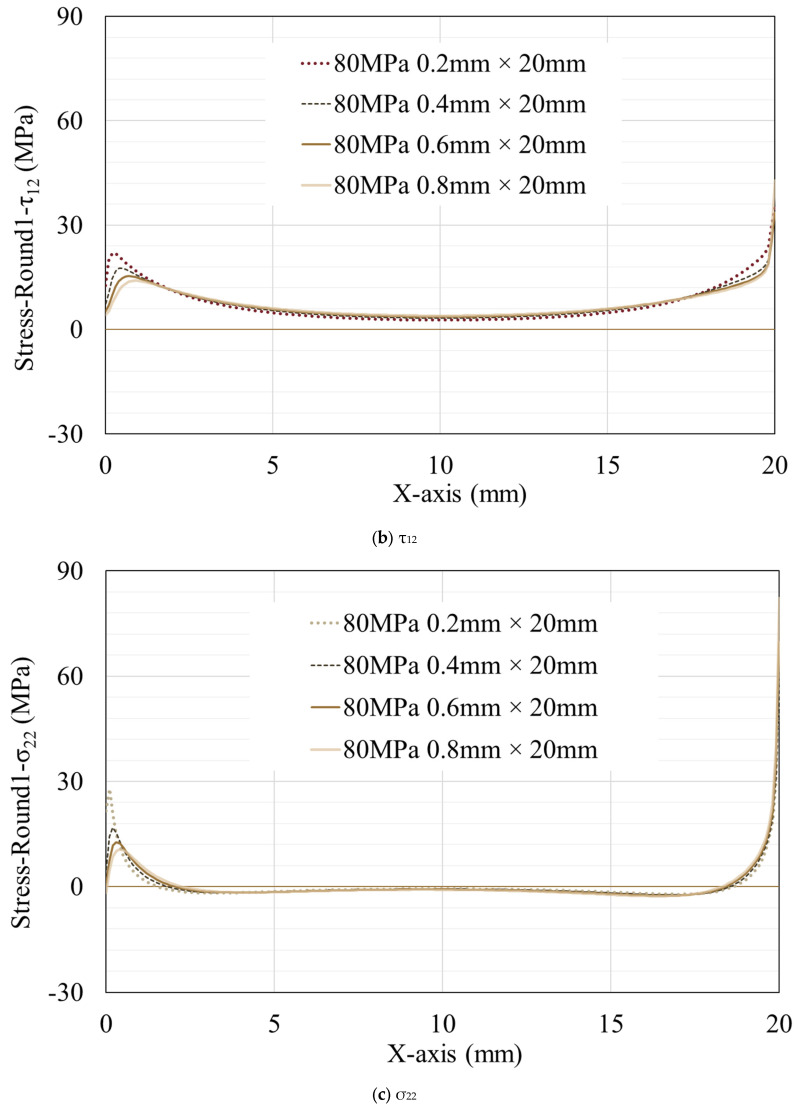
Stress distribution on the surface of resins with different thicknesses after the first tension. The data extraction locations are indicated by the red line in Figure 3.

**Figure 8:**
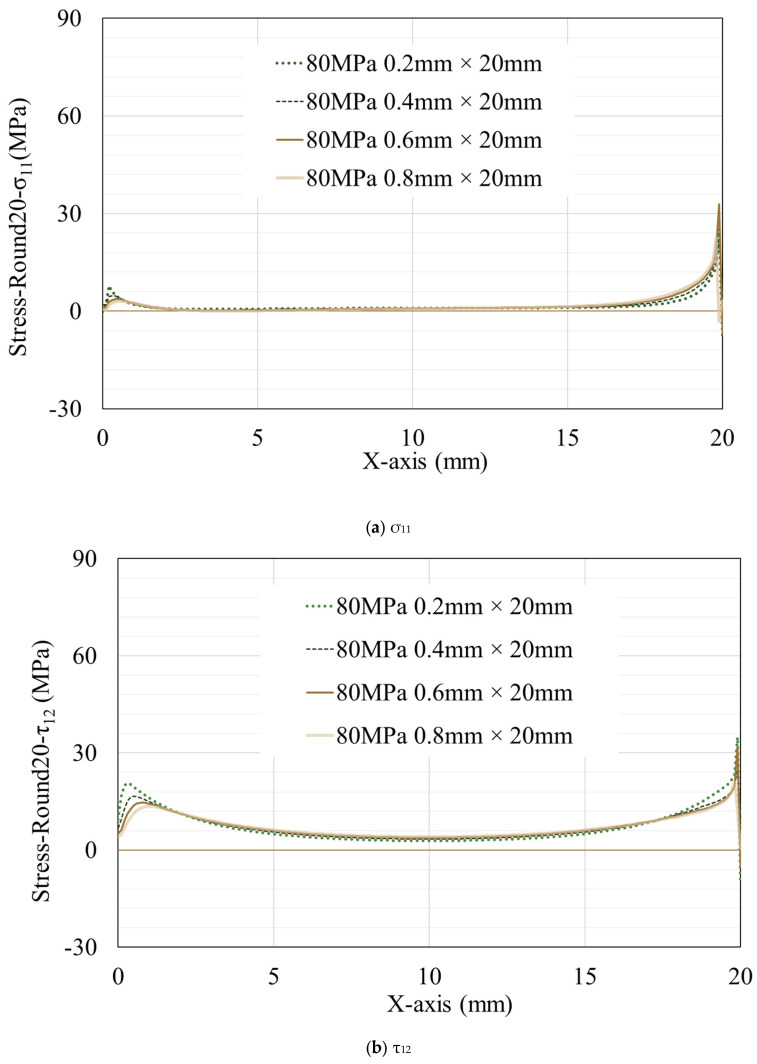

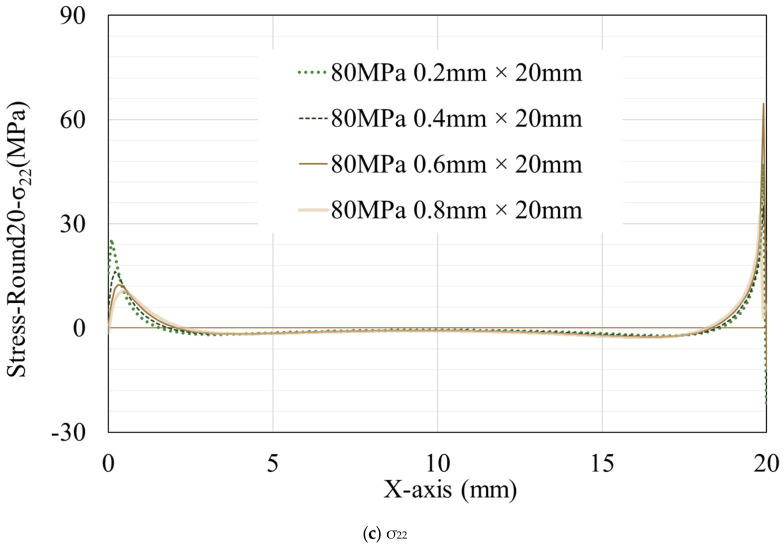
Stress distribution on the surface of resin with different thicknesses after the 20th tension cycle. The data extraction locations refer to the red line in Figure 3.

**Figure 9:**
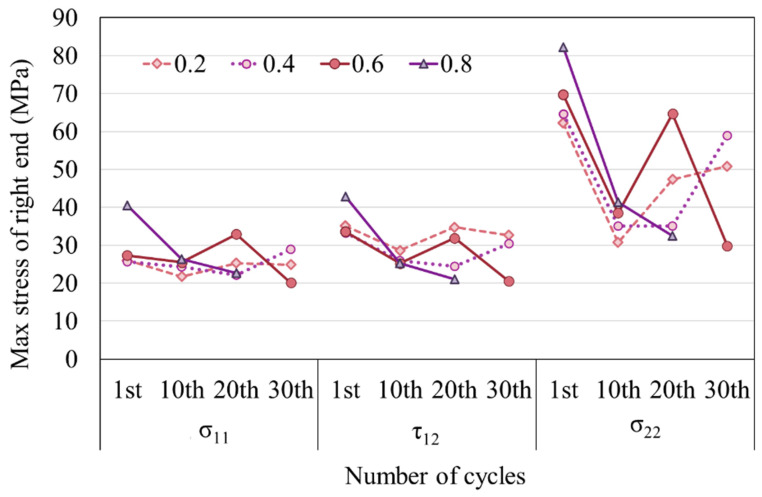
Relationship between the peak stress on the right side of the resin and thickness.

To discuss the stress distribution in resins of different thicknesses under an equal number of tension cycles, data from models with thicknesses of 0.2 mm, 0.4 mm, 0.6 mm, and 0.8 mm at the end of the 1st and 20th tension cycles are selected for comparison (only regular hexahedral elements are selected here) (Figure 7). It can be observed that stress peaks appear at both ends of the resin. The stress peak on the left side is inversely proportional to the resin thickness and remains nearly constant throughout the tensile simulation (for clarity in the illustration, not all data are chosen, but the simulation results indicate that, in the case of 0.1 mm thickness, the peak values of σ_11_, τ_12_, and σ_22_ on the left are almost as high as, or even surpass, those on the right). As mentioned earlier, although the peak stress at the right end varies with the increase in the number of tension cycles, in the first tension cycle, the models with thicker resins generally exhibit higher stress peaks (Figure 8). As tension progresses, the models with thicker resins experience a more rapid shift in stress peaks, and the stress at the terminal elements decrease more rapidly. This leads to the fluctuation of the peak values on the right side (Figure 9) during the 10th, 20th, and 30th cycles. The peak values at these times are not simply related to the thickness of the resin.

### 3.3. Relationship between Number of Cycles to Failure (N_f_) and Thickness

**Figure 10:**
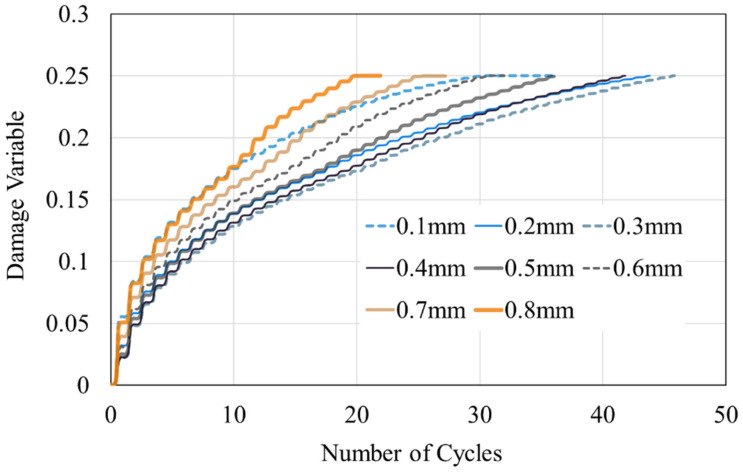
Variation in the damage variables of the upper-right element under different resin thicknesses with increase in number of tension cycles.

**Figure 11:**
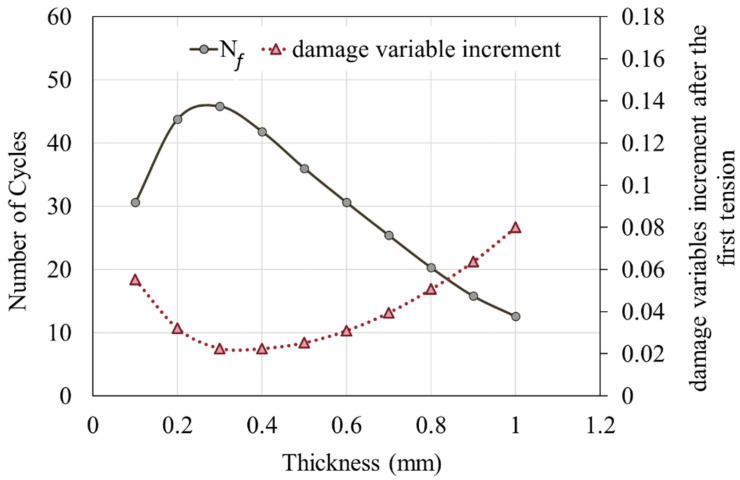
Relationship between the number of cycles to failure, damage variable increment after the first tension, and resin thickness.

A comparison of the damage variable of the outermost element at the top right of the resin reveals that as the thickness increases, the number of tensile cycles required for the damage variables to attain the failure threshold of 0.25 (N_f_) first increases and then decreases. The model with a resin thickness of 0.3 mm results in the maximum N*_f_* value, as shown in Figure 10. As is evident from the graph, the damage accumulation from the first tension is greater than the damage accumulated from subsequent tensions. The increase in damage variable after the first tension is indicative of the failure tendencies of the models with varying thicknesses. From Figure 10, the relationship between the damage variable, N*_f_*, and thickness has been collated to Figure 11. As the resin thickness increases, N*_f_* initially increases and subsequently decreases. Conversely, the increment in damage variable after the first tension exhibits an inverse pattern: for resin thicknesses below 0.3 mm, it reduces with an increase in the thickness, whereas for thicknesses beyond 0.3 mm, the increment increases. The relationship between N*_f_* and the increment in damage variable after the first tension suggests that reducing the increment in damage variable after the first tension may extend the lifespan of the model.

These observations indicate the existence of an optimal adhesive thickness that maximizes the durability of adhesive joints. However, the reason for the peak performance at this particular thickness remains unclear. Whether the optimal adhesive thickness (0.3 mm) applies to models with different adhesive layer lengths and which other factors may influence the ideal adhesive thickness still need to be investigated.

## 4. Conclusions

This study discusses the stress distributions and fatigue behaviors of SLS models with different adhesive layer thicknesses. The key points are summarized below:

(1) For the first failing regular, hexahedral resin element in the single-lap shear model: during tension, the value of σ_22_ on the resin element is larger than both τ_12_ and σ_11_. The order of magnitude between τ_12_ and σ_11_ varies depending on the thickness of the resin. Additionally, as the remaining life approaches half its lifespan, τ_12_ experiences a larger reduction than σ_11_.
(2) Stress peaks appear at both ends of the resin across the adhesive interface under tension. The values of left-side stress peaks are related to the resin thickness and remain consistent throughout the tension simulation. In contrast, the right-side stress peaks are positively correlated with the resin thickness only during the first tension; subsequently, their values and positions change with the increase in the number of tensile cycles.
(3) With an increase in the resin thickness, N*_f_* initially increases and then decreases. The model with a resin thickness of 0.3 mm achieves the longest lifespan. Meanwhile, the increase in the damage variable after the first tension exhibits an opposing trend.

In this study, the optimal resin thickness for damage resistance is approximately 0.3 mm. These conclusions provide valuable insights into the resin–CFRP interface stress behavior and resin failure mechanisms under cyclic loading.

## Figures and Tables

**Table 1:** Property parameters of CFRP [44].

E_1_	E_2_	E_3_	Nu_12_	Nu_13_	Nu_23_	G_12_	G_13_	G_23_
130,000	9530	9530	0.34	0.34	0.4	4730	4730	3180

**Table 2:** Property parameters of resin [40].

*n*	$E_{n}\left(MPa\right)$	$\eta_{n}(MPa\cdot s)$	Elasticity	
1	284	4.5 × 10^2^	$E_{0}\left(\mathrm{MP}a\right)$	4260
2	284	3.3 × 10^3^	ν	0.3
3	284	1.2 × 10^5^	Nonlinearity	
4	284	1.9 × 10^6^	$\sigma_{0}\left(\mathrm{MP}a\right)$	70
5	284	1.8 × 10^7^	α	2
6	284	1.4 × 10^8^	*m*	7
7	284	8.5 × 10^8^	Viscoplastic strain	
8	284	5.0 × 10^9^	$\eta_{0}\left(\mathrm{MP}a\cdot s\right)$	1.0 × 10^23^
9	284	3.0 × 10^10^	$\sigma_{vp\_0}\left(\mathrm{MP}a\right)$	0
10	284	1.9 × 10^11^	$\alpha_{vp}$	0
11	284	1.4 × 10^16^	$\beta_{vp}$	0
12	284	1.3 × 10^19^	*χ*	0
13	284	2.1 × 10^22^		
14	284	1.3 × 10^26^	Damage variables	
15	284	2.5 × 10^29^	$\alpha_{d}$	4

## Data Availability

The data that support the findings of this study are available from the corresponding author upon reasonable request.

## Nomenclatures

ε, Δε	Total strain tensor, increment in total strain tensor
$\varepsilon^{ve}$, $\varepsilon^{vp}$	Viscoelasticity strain tensor, viscoplasticity strain tensor
σ, Δσ, s	Stress tensor, increment in stress tensor, deviatoric stress tensor
p, I	Hydrostatic stress, second-order identity tensor
D, $D_{cr}$	Damage variable, critical damage variable
s, $s^{f}$	Entropy generation, final fracture entropy
g	Nonlinear coefficient
t, $t^{\prime }$	Time
W	Dissipated energy
$\alpha_{d}$	Damage parameters
$E^{r}$	Relaxation tensor
H	Viscosity matrix
M	Constant matrix
D, $E_{0}$, ν	Stiffness matrix, initial Young’s modulus, Poisson’s ratio
$E_{n}$ $\eta_{n}$	Viscoelasticity properties of Maxwell elements
$\sigma_{eqv}$, $\varepsilon_{eqv}^{vp}$	Equivalent stress, equivalent viscoplastic strain
$\eta^{vp}$, $\eta_{0}$, α, β, χ	Viscoplasticity coefficients
A, V	Generalized thermodynamic force and internal flow vectors
T, Q	Temperature, heat flux vector
